# Selective Oxidation of α,β‐Unsaturated Alcohols With Lyophilisates of *Bjerkandera adusta*

**DOI:** 10.1002/cbdv.202501127

**Published:** 2025-08-18

**Authors:** Letizia Calza, Valeriia Nikitenkova, Wendell Albuquerque, Holger Zorn, Tatyana Zhuk

**Affiliations:** ^1^ Institute of Food Chemistry and Food Biotechnology, Justus Liebig University Giessen Giessen Germany; ^2^ University of Modena and Reggio Emilia Modena Italy; ^3^ Fraunhofer Institute for Molecular Biology and Applied Ecology Giessen Germany; ^4^ Department of Organic Chemistry Igor Sikorsky Kyiv Polytechnic Institute Kyiv Ukraine

**Keywords:** alcohol oxidation, allylic aldehydes, flavor compounds, whole cells

## Abstract

The biocatalytic aerobic production of (*E*)‐2‐allylic aldehydes from their corresponding alcohols using lyophilisates of the basidiomycetous fungus *Bjerkandera adusta* is reported. The addition of small amounts of organic solvents to the reaction media increased the reaction and substrate conversion rates, allowing for to produce (*E*)‐aldehydes under sustainable conditions. Citral (mixture of (*E*)‐ and (*Z*)‐3,7‐dimethylocta‐2,6‐dienal) was found as a result of the oxidation of geraniol ((*E*)‐3,7‐dimethyl‐2,6‐octadien‐1‐ol) as well as of nerol ((*Z)*‐3,7‐dimethyl‐2,6‐octadien‐1‐ol). In the case of (*Z*)‐2‐nonene‐1‐ol, the formation of (*E*)‐2‐nonenal was detected as the only product. The isomerization of (*Z*)‐2‐nonenal to the corresponding (*E*)‐diastereomer with fungal lyophilisate under the applied reaction conditions has been shown in additional experiments. No products of further oxidation of the formed aldehydes or reduction of the activated CC‐double bond were detected in the reaction mixtures. A comparative analysis of the catalytic activity of lyophilisates obtained from *Pleurotus sapidus* and *Pleurotus eryngii*, as well‐known producers of oxidative enzymes, was performed in model experiments.

## Graphical abstract

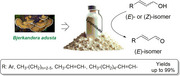

## 1 Introduction

α,β‐Unsaturated aldehydes are well‐known building blocks in organic synthesis [1, 2, 3] as well as flavor compounds used in food and beverages [4]. They are responsible for a wide variety of odor impressions, from the fresh green notes of (*E*)‐2‐hexenal to the fatty, chicken‐like flavor of (*E,E*)‐2,4‐decadienal. Despite their widespread occurrence in nature, especially in plants [5], they are not accumulated in organisms but rather serve as cosubstrates for *in vivo* enzymatic transformations [6]. For this reason, rich natural sources of aldehydes are rare.

**FIGURE 1:**
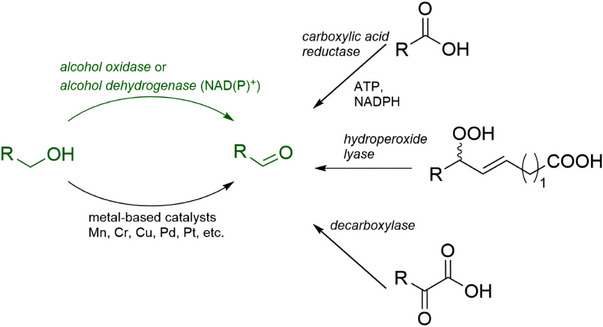
Selected biocatalytic approaches for aldehyde production (ATP – adenosine triphosphate; NADPH – nicotinamide adenine dinucleotide phosphate). These cofactors are required in stoichiometric amounts.

A number of chemical methods for the synthesis of α,β‐unsaturated aldehydes have been developed [7, 8, 9]. However, even mild approaches are based on the utilization of toxic metals, which makes them inappropriate for using the produced aldehydes in food, beverages, and cosmetics. Recently, biocatalytic transformations attracted much attention as promising sustainable alternatives to traditional chemical approaches [10]. Nature developed a portfolio of proteins to transform different types of precursors to target aldehydes, and many researchers attempt their application at laboratory and industrial scale [11, 12, 13, 14]. The simplest and most common method for the preparation of aldehydes is based on the oxidation of primary alcohols that, in many cases, suffer from overoxidation, sensitivity to the alkyl chain length, long reaction times, as well as low conversion and space–time yields [10]. Current biocatalytic approaches are mainly based on the utilization of alcohol oxidases [15, 16], alcohol dehydrogenases [17, 18], carboxylic acid reductases [19, 20], decarboxylases (for α‐keto acid decarboxylation) [21, 22], and the combination of lipoxygenases with hydroperoxide lyases [23] (Figure 1). Alcohol dehydrogenases and carboxylic acid reductases are well‐studied catalysts; however, they require the presence of stoichiometric amounts of expensive cofactors or the implementation of cofactor regeneration systems. Decarboxylases and hydroperoxide lyases are appropriate for specific substrates, namely ketoacids and hydroperoxides of polyunsaturated fatty acids, respectively. Aryl alcohol oxidases, which are FAD (flavin adenine dinucleotide)‐dependent oxidoreductases, seem to be the most promising biocatalysts as they require only molecular oxygen as a cosubstrate for alcohol oxidation and do not depend on costly external cofactors [24, 25]. Alcohol oxidases have been successfully applied for the synthesis of furan‐based derivatives [26, 27, 28], vanillin [29], and for the oxidation of methanol and glycerol [30].

Currently, the main research activities on biocatalytic approaches are associated with the construction of recombinant expression systems for target enzyme production, followed by enzyme purification, which is still economically challenging. Recently, the examination of the dependency of the manufacturing costs of whole‐cells *vs*. enzymes showed that prices for industrial (non‐purified) enzymes range between 250 and 1000 €*kg^−1^, whereas whole‐cells are considerably cheaper (35–100 €*kg^−1^) [31]. The main efforts in whole‐cell production are focused on the implementation of modified host organisms, but there are many potential industrial applications in which it would be more desirable to avoid the handling of living genetically modified systems.

An alternative protocol for the production of aromatic aldehydes from the corresponding alcohols using *Bjerkandera adusta* (BAD) lyophilisate has been described by us recently [32]. The approach allows overcoming the challenges of recombinant expression systems and enzyme purification, as well as uses the main advantages of whole‐cells, namely low cost, enzyme stability, and independence from additional cofactors. The high catalytic activity of BAD lyophilisate is associated with the confirmed presence of aryl‐alcohol oxidases that preserve their catalytic activity after the freeze‐drying process and long‐term storage. One of the main challenges of the application of this approach to the oxidation of allylic alcohols could be the presence of an activated CC‐double bond in the substrates, which may be reduced easily, unlike the aromatic ring of benzyl alcohol. Previously, the ability to reduce CC‐double bonds in α,β‐unsaturated substrates was shown for various fungi from the Phylum Basidiomycota [33, 34].

The current study aims to explore the prospects of BAD lyophilisates to oxidize allylic alcohols to the corresponding carbonyl compounds. The scope and limitations of allylic alcohol oxidation depending on their molecular structures are disclosed as well.

## 2 Results and Discussion

### 2.1 Influence of the pH and Presence of Cosolvents

**FIGURE 2:**
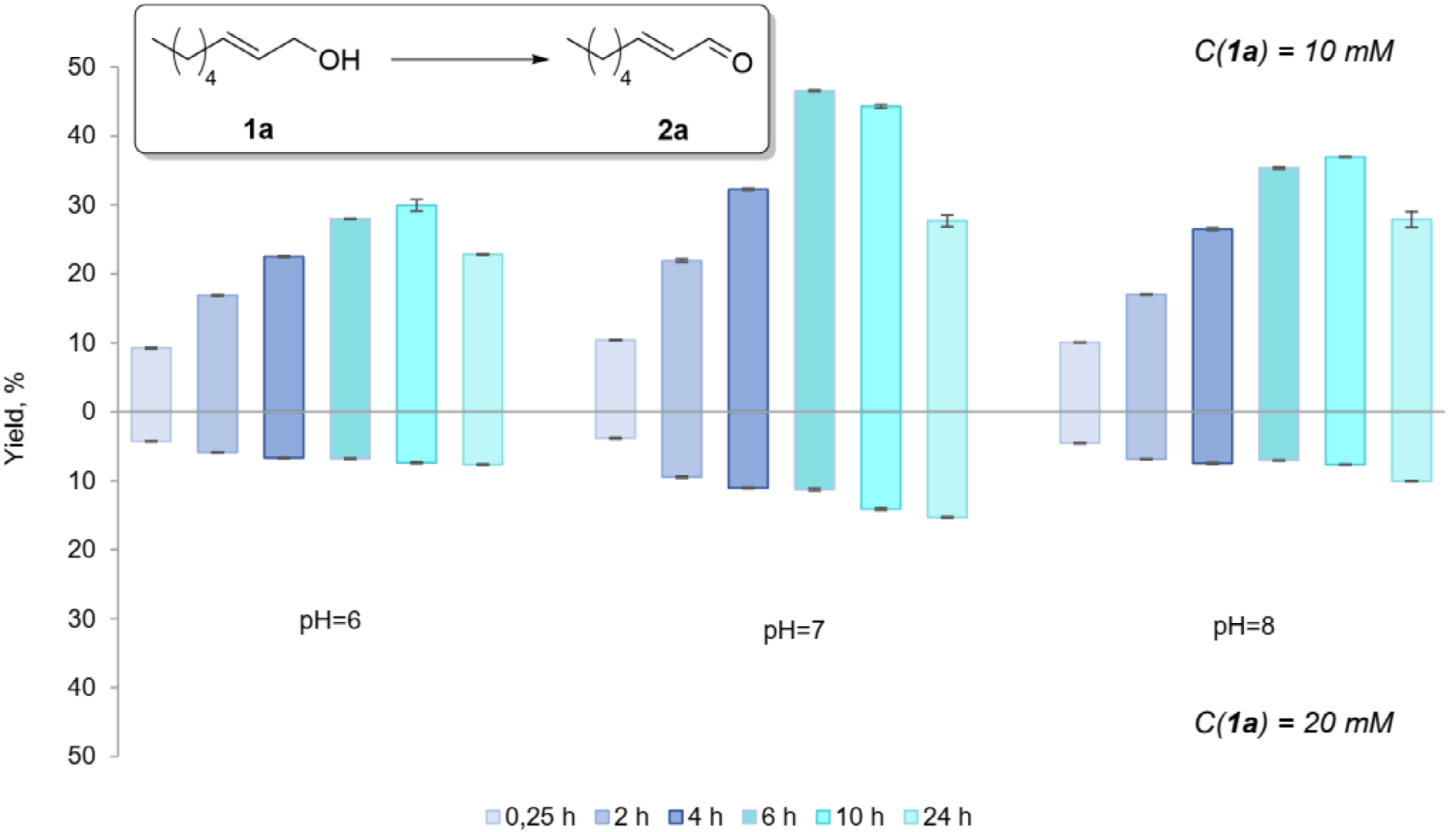
Yields of (*E*)‐2‐octenal (**2a**) after 15 min, 2 h, 4 h, 6 h, 10 h, and 24 h at pH 6, 7, and 8. The initial concentrations of the substrate (*E*)‐2‐octen‐1‐ol (**1a**) were 10 mM (upper part) and 20 mM (bottom part).

The oxidative activity of BAD lyophilisates towards (*E*)‐2‐octen‐1‐ol (**1a**) as a model compound was estimated using the procedure developed previously for aromatic alcohols [32]. The biotransformation of **1a** was studied at pH 6, 7, and 8, which are optimal for the enzymatic oxidation of alcohols, as previously described [32, 35]. The conversion of the alcohol to the aldehyde was analyzed after 15 min, 2, 4, 6, 10, and 24 h. (Figure 2) Product concentrations were determined utilizing gas chromatography‐mass spectrometry (GC‐MS) via an internal standard (1‐adamantanol). Calibration curves were established for the target aldehydes using commercially available standards. Notably, after 15 min, the yield of aldehyde **2a** already reached 10% in all experiments. The content of **2a** reached maximum values between 6 and 10 h of reaction time. Among the tested pH values, the highest yield (up to 45%) was observed at pH 7. Decreasing the concentration of the substrate increased the yield; thus, at pH 7, experiments with 10 mM of (*E*)‐2‐octen‐1‐ol (**1a**) gave a higher yield of aldehyde **2a**, namely up to 45 % compared to 15 % with 20 mM. Notably, the reaction is remarkably selective as **2a** was observed as the only product. The simplicity of the work‐up of the reaction mixture by simple filtration/extraction opens certain perspectives for this methodology. However, high conversion rates of starting alcohol **1a** were not reached through the applied “on‐water” methodology, and attention was thus turned to the use of organic cosolvents.

**FIGURE 3:**
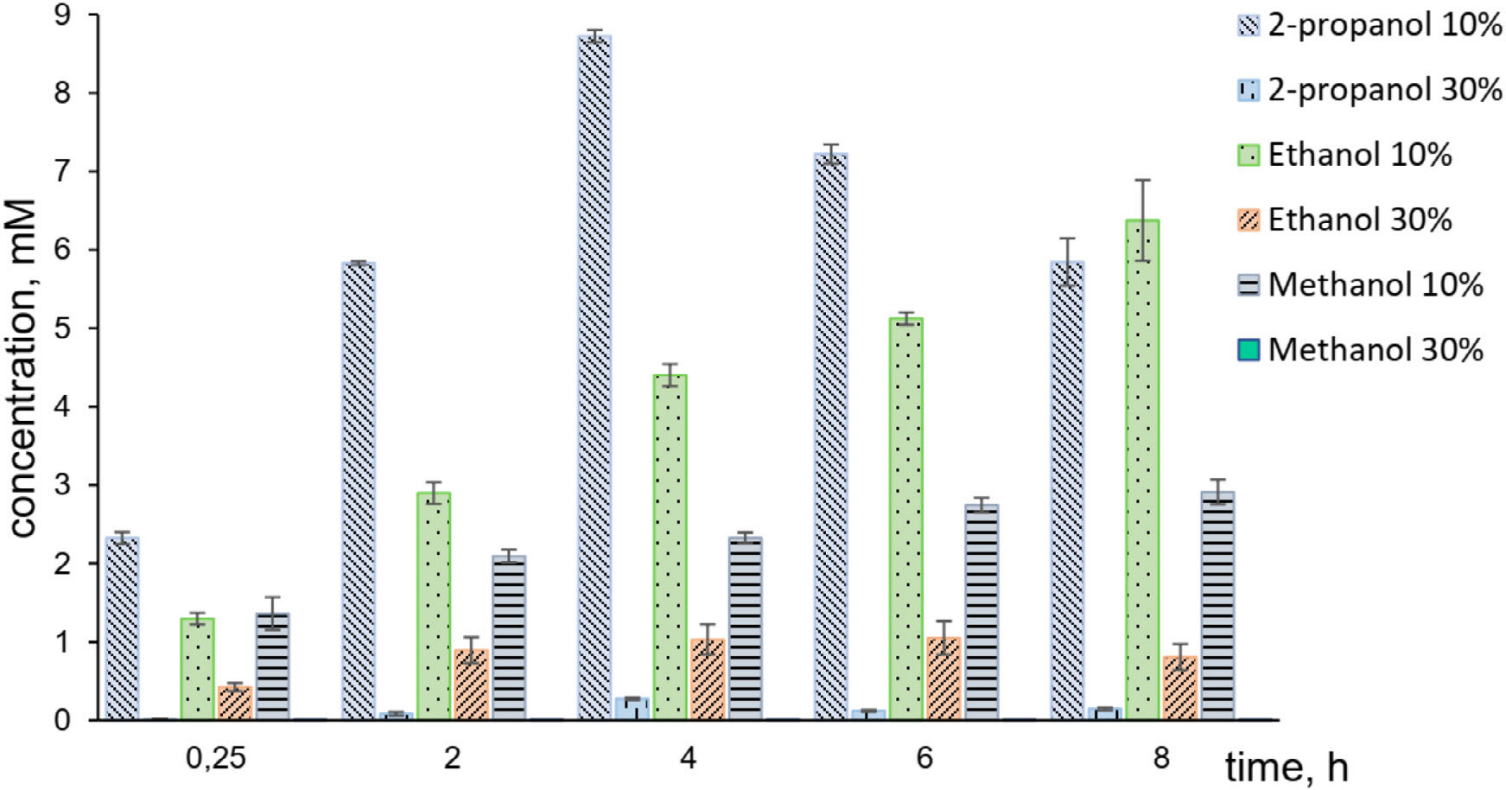
Concentration of (*E*)‐2‐octenal (**2a**) as a product of biotransformation of alcohol **1a** (10 mM) performed in the presence of different cosolvents (methanol, ethanol, and 2‐propanol) in concentrations of 10% and 30%.

Methanol, ethanol, and isopropanol were tested as cosolvents in concentrations of 10 and 30 volume % (Figure 3). The presence of 30 % of every solvent decreased the conversion of substrate **1a** significantly, likely due to enzyme denaturation at such high cosolvent concentrations. In contrast, with 10 % of cosolvent, the reaction proceeded smoothly, suggesting that the enzyme remained stable under these milder conditions. Among the tested alcohols, isopropanol proved to be the most effective, likely due to its moderate polarity and relatively bulky structure, which together provided a favorable balance between enzyme stability and substrate solubility. Under these conditions, a ca. 90 % yield of **2a** was reached after 4 h. This yield was confirmed by performing preparative experiments, in which (*E*)‐2‐octenal (**2a**) was isolated and characterized through nuclear magnetic resonance (NMR) spectroscopy. These reaction conditions were further used for the study of the substrate scope. In control reactions with autoclaved lyophilisates, only the substrate **1a,** without any traces of products, was detected in the reaction mixtures.

### 2.2 Biotransformation of α,β‐unsaturated Primary Alcohols

After optimization of the reaction conditions using (*E*)‐2‐octen‐1‐ol (**1a**), the activity of BAD lyophilisates was tested toward a number of substrates of different structures (Table 1). Product formation was monitored utilizing GC‐MS with an internal standard (1‐adamantanol). For substrates **1a** and **1d–i**, the oxidation reactions were performed on a preparative scale, and the products were isolated using column chromatography. (*E*)‐2‐Nonen‐1‐ol (**1b**) and (*E*)‐cinnamyl alcohol (**1c**), as well as model alcohol **1a**, were fully converted to the corresponding aldehydes **2b** and **2c**. (*E*)‐2‐Hexen‐1‐ol (**1d**), which contains a shorter carbon chain, demonstrated low conversion, giving only 20% of (*E*)‐2‐hexenal (**2d**) while the yield of (*E,E*)‐2,4‐hexadienal (**2e**) from the corresponding alcohol **1e** reached 65%. With *(E,E*)‐2,4‐decadien‐1‐ol (**1f**), traces of diastereomeric aldehyde **3f** were identified, together with the main product *(E,E*)‐2,4‐decadienal (**2f**) with 68% yield. Citral (3,7‐dimethyl‐2,6‐octadienal, **2g**), which is a mixture of geranial and neral [36, 37], was found as a product of the oxidation of geraniol (**1g**) as well as of nerol (**1h**). However, the conversion of nerol (**1h**) reached only 57%, while geraniol (**1g**) was fully converted. Notably, the oxidation of (*Z*)‐2‐nonen‐1‐ol (**1i**) gave selectively (*E*)‐2‐nonenal (**2b**), the product of isomerization without any traces of the *Z*‐configured aldehyde, with a conversion of the substrate of 86%. No oxidation products were found with 1‐hepten‐3‐ol (**1j**), 1‐nonen‐3‐ol (**1k**), 1‐octyn‐3‐ol (**1l**), and cyclohexen‐1‐ol (**1m**). These findings clearly demonstrate the selectivity of the transformation of primary alcohols. Since possible by‐products, such as the respective acids or starting/saturated alcohols, could either be adsorbed by the lyophilisate or be highly water‐soluble, preparative experiments with selected alcohols ((*E*)‐2‐hexen‐1‐ol (**1d**), *(E,E*)‐2,4‐hexadien‐1‐ol (**1e**), *(E,E*)‐2,4‐decadien‐1‐ol (**1f**), nerol (**1h**), geraniol (**1g**), and (*Z*)‐2‐nonen‐1‐ol (**1i**)) were performed displaying high reaction yields.

**TABLE 1:** Biotransformation of α,β‐unsaturated alcohols.^*^

#	Substance		Conversion, %	Product		Yield, %
1	(*E*)‐2‐octen‐1‐ol (**1a**)	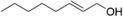	100	(*E*)‐2‐octenal (**2a**)	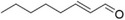	87^§^
2	(*E*)‐2‐nonen‐1‐ol (**1b**)	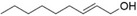	100	(*E*)‐2‐nonenal (**2b**)	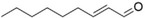	99
3	(*E*)‐cinnamyl alcohol (**1c**)	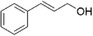	100	(*E*)‐cinnamaldehyde (**2c**)	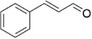	99
4	(*E*)‐2‐hexen‐1‐ol (**1d**)	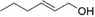	23	(*E*)‐2‐hexenal (**2d**)	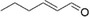	20^§^
5	*(E,E*)‐2,4‐hexadien‐1‐ol (**1e**)	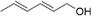	73	*(E,E*)‐2,4‐hexadienal (**2e**)	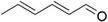	65^§^
6	*(E,E*)‐2,4‐decadien‐1‐ol (**1f**)	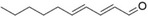	84	*(E,E*)‐2,4‐decadienal (**2f**) Diastereomeric 2,4‐decadienal (**3f**)	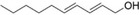	68^§^ traces
7	Geraniol (**1g**)	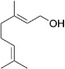	100	Citral (**2g**)^a^	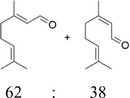	95^§^
8	Nerol (**1h**)	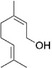	57	Citral (**2g**)^a^	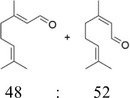	52^§^
9	(*Z*)‐2‐nonen‐1‐ol (**1i**)	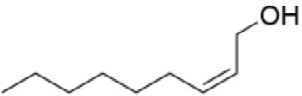	86	(*E*)‐2‐nonenal (**2b**)	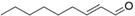	83^§^
10	1‐hepten‐3‐ol (**1j**)	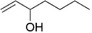	0	—		0
11	1‐nonen‐3‐ol (**1k**)	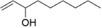	0	—		0
12	1‐octyn‐3‐ol (**1l**)	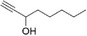	0	—		0
13	2‐cyclohexen‐1‐ol (**1m**)	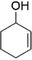	0	—		0

^a^ Determined by GC‐MS from relative peak areas.

* All reactions were performed under identical conditions as described in the Experimental Section 3.6 (General procedure for biotransformation).

^§^ Isolated yields.

### 2.3 Biotransformation of (*Z*)‐/(*E*)‐2‐nonen‐1‐ol

**FIGURE 4:**
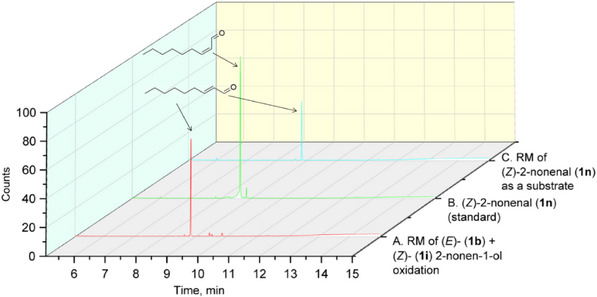
Gas chromatography‐mass spectrometry (GC‐MS) chromatograms of: (A) The reaction mixtures (RM) of biotransformation of an equimolar mixture of (*Z*)‐ (**1i**) and (*E*)‐ (**1b**) 2‐nonen‐1‐ols; (B) Standard of (*Z*)‐2‐nonenal (**1n**); (C) The reaction mixtures (RM) of the transformation of (*Z*)‐2‐nonenal (**1n**).

For geraniol (**1g**), nerol (**1h**), and (*Z*)‐2‐nonen‐1‐ol (**1i**), the partial or full conversion of one geometric isomer to another has been observed. It is likely that amino acids, acting as zwitterions, contribute to the stabilization of intermediates during the isomerization process. The catalytic ability of several amino acids, as well as of bovine serum albumin, in promoting the isomerization of geranial and neral has already been demonstrated [38]. Notably, neither the presence of vitamin C nor conducting the reaction under an inert atmosphere affected the isomerization rate. As the fungal lyophilisates contain numerous proteins, the reaction conditions were tested using *(Z)*‐2‐nonenal (**1n**), which was synthesized using Dess‐Martin periodinane from *(Z)‐*2‐nonen‐1‐ol (**1i**). Under standard reaction conditions with BAD lyophilisate, *(Z)*‐2‐nonenal (**1n**) gave (*E*)‐2‐nonenal (**2b**) as the only product. (Figure 4) This result is in accordance with the above‐mentioned isomerization of geranial and neral.

### 2.4 Characterization of Further Fungal Lyophilisates

**FIGURE 5:**
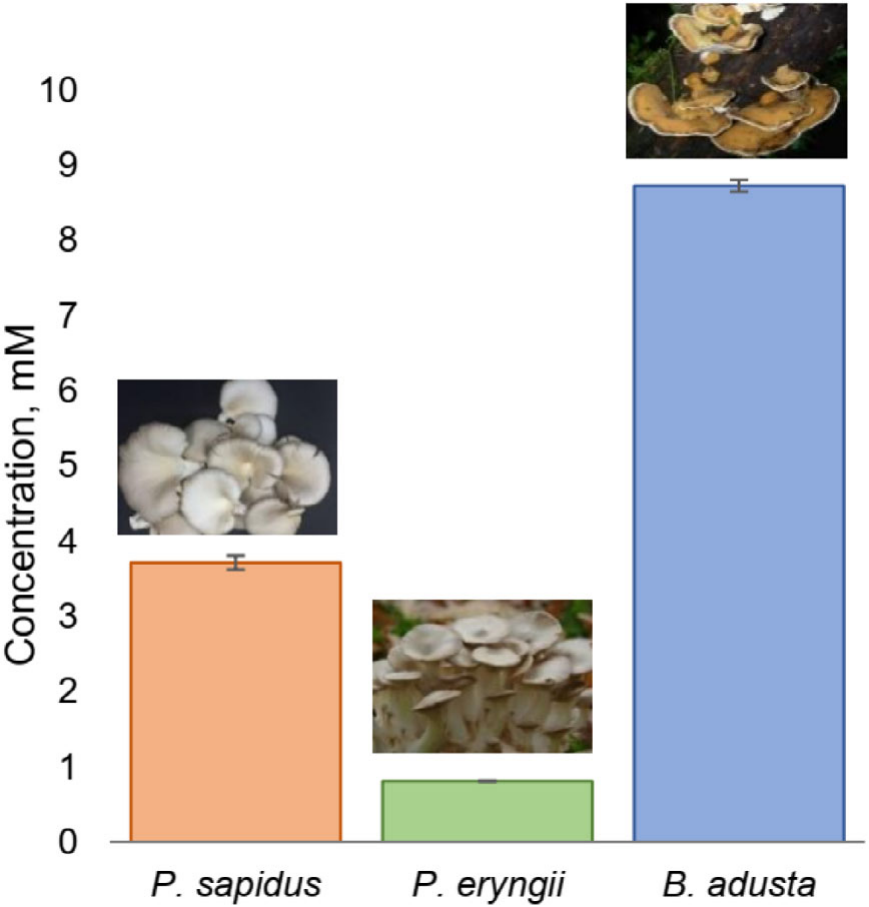
Comparative analysis of oxidation of (*E*)‐2‐octen‐1‐ol (**1a**) with lyophilisates of *P. sapidus, P. eryngii*, and *B. adusta*.

*Pleurotus eryngii* and *Pleurotus sapidus* are known as sources of oxidative enzymes. As only supernatants of the corresponding fungi have been well studied for the presence of oxidative enzymes, the ability of their lyophilisates to catalyze allylic alcohol oxidation reactions similar to BAD lyophilisates was investigated. The standard procedure for lyophilisate production and (*E*)‐2‐octen‐1‐ol (**1a**) as a model compound was used (Figure 5). The results showed that these lyophilisates are not as efficient as those from BAD, but have some potential as well. In particular, while the lyophilisate of *P. eryngii* gave low conversions of the starting compounds, the lyophilisates of *P. sapidus* displayed certain activity, still much lower than that observed for the BAD lyophilisate. The activities of *P. eryngii* and *P. sapidus* lyophilisates were studied under the conditions that are the most efficient for BAD lyophilisate, thus giving a rough estimation of their activity. By varying the cultivation time and conditions, as well as cosolvent and pH values, it could be possible to increase their catalytic activity. However, the developed simple conditions clearly show the advantage of the BAD lyophilisate.

## 3 Experimental

### 3.1 Organism

The filamentous fungi BAD (DSMZ 4708)*, P. eryngii* (DSMZ 8264), and *P. sapidus* (DSMZ 8266) were obtained from the German Collection of Microorganisms and Cell Cultures, Brunswick, Germany. The stock cultures were maintained on a solid medium containing 15 g/L malt extract (Fluka, Neu‐Ulm, Germany) and 15 g/L agar‐agar (Roth, Karlsruhe, Germany).

### 3.2 Chemicals

1‐Adamantanol (99%), *(E)‐*2‐nonen‐1‐ol (96%), 2‐cyclohexen‐1‐ol (95%), Dess‐Martin periodinane (97%), and *(E)*‐2‐nonenal were obtained from Sigma‐Aldrich (St. Louis, Missouri, USA). 1‐Hepten‐3‐ol (98%), 1‐nonen‐3‐ol (98%), 1‐octyn‐3‐ol (98%), 2,4‐hexadien‐1‐ol (99%), *(Z)*‐2‐hexen‐1‐ol (94%), *(E)*‐2‐octen‐1‐ol (97%), citral (*E+Z*, 95%), and *(E,E)*‐2,4‐hexadienal (95%) were purchased from Alfa Aesar, Thermo Fisher (Kandel, DE). 2,4‐Decadien‐1‐ol (95%) and *(Z)*‐2‐nonen‐1‐ol (95%) were obtained from abcr GmbH (Karlsruhe, DE). Cinnamyl aldehyde (98%) and *(E)*‐2‐hexen‐1‐ol (97%) were purchased from Carl Roth GmbH & Co. KG (Karlsruhe, DE). Geraniol (99%), nerol (97%), *(E)*‐2‐hexenal (99%), and *(E)*‐2‐cinnamyl alcohol (98%) were bought from Acros Organics (New Jersey, USA). *(E)*‐2‐Octenal (98%) was purchased from BLD Pharmatech GmbH (Reinbek, DE). *(E,E)*‐2,4‐Decadienal (95%) was obtained from Fisher Scientific GmbH (Schwerte, DE).

### 3.3 NMR and MS Instrumentation

The following instruments were used during this study: Rotavapor from Heidolph (Schwabach, Germany) for the evaporation procedure; T 25 digital Ultra‐Turrax homogenizer (IKA, Staufen, Germany) for the homogenization of submerged cultures; shaker (Orbitron, Infors HAT, Bottmingen, Switzerland) for the incubation of cultures, freeze dryer Vaco2 (Zirbus Technology, Bad Grund, Germany) for lyophilization of mycelium.

NMR spectra were recorded at 298 K on a Bruker Avance II 400 MHz WB instrument (Billerica, Massachusetts, USA). The CHCl_3_ signal (s, 7.26 ppm) was set as reference for the spectra in CDCl_3_. NMR spectra were reported in Figures S1–S14.

GC‐MS was performed using an Agilent 7890A gas chromatograph equipped with an Agilent VF‐WAXms capillary column (30 m × 0.25 mm, 0.25 µm) and an Agilent 5975C MSD Triple‐Axis mass spectrometer.

### 3.4 Lyophilized Mycelium

The culture medium was prepared by dissolving malt extract (20 g) in 1 L of deionized water. For the preparation of the precultures, a 1 cm^2^ agar plug from the leading mycelial edge was transferred into 100 mL medium (in a 250 mL Erlenmeyer flask) and homogenized with a T 25 digital Ultra‐Turrax homogenizer (IKA, Staufen, Germany; 30 s, 10.000 r⋅min^−1^). The precultures were grown on an incubation shaker (Orbitron, Infors HAT, Bottmingen, Switzerland; 150 r⋅min^−1^, deflection 25 mm) under the exclusion of light at 24°C for 7 days. Afterwards, the precultures were homogenized, and 10% (v/v) of the homogenate was inoculated for submerged cultivation on a 400 mL (in 1 L Erlenmeyer flasks) scale. After 6 days of growth in submerged culture, the mycelium was harvested by filtration and washed with distilled water. The obtained biomass was freeze‐dried (Vaco2, Zirbus Technology, Bad Grund, Germany) and stored at ‐20°C until usage.

### 3.5 Influence of the pH Value on the Biotransformation

BAD lyophilisate (320 mg) was rehydrated in 20 mL potassium phosphate buffer (100 mM) at different pH values ranging from 6.0 to 8.0 (24°C) by stirring at 650 rpm for 10 min. (*E*)‐2‐Octen‐1‐ol (**1a**) was added in different concentrations (10 mM and 20 mM), and the reaction mixture was further stirred for 24 h. Samples were taken after 15 min, 2 h, 4 h, 6 h, 10 h, and 24 h. For analysis, 1 mL of the reaction mixture was taken, the pH was adjusted to 7, and sodium chloride (100 mg) was added to the reaction mixture. 1 mL of pentane containing 1‐adamantanol (8 mM) was added as an internal standard, and the resulting mixture was shaken and centrifuged (4000 *× g*, 2 min, 4°C) to separate the phases. The extraction process was repeated three times. The combined organic phases were dried over Na_2_SO_4_ and analyzed by GC‐MS. Blank transformations were performed with autoclaved lyophilisates as controls. Every experiment was performed in triplicate.

### 3.6 Influence of Organic Cosolvents on the Biotransformation

BAD lyophilisate (320 mg) was rehydrated in 18 mL (10% of cosolvent) or 14 mL (30% of cosolvent) potassium phosphate buffer (100 mM, pH = 7) by stirring at 650 rpm at 24°C for 10 min. (*E*)‐2‐Octen‐1‐ol (**1a**) was added to the reaction mixture as a solution in 2 mL (10% of cosolvent) or 6 mL (30% of cosolvent) of organic solvent to a final concentration of 10 mM. The reaction mixture was stirred at 24°C and 650 rpm for 8 h. Samples were taken after 15 min, 2 h, 4 h, 6 h, and 8h. The samples were processed as described in section 3.5.

### 3.7 General Procedure for Biotransformation

BAD lyophilisate (320 mg) was rehydrated in 18 mL potassium phosphate buffer (100 mM, pH = 7) by stirring at 650 rpm at 24°C for 10 min. The substrate was added to the reaction mixture as a solution in 2 mL of 2‐propanol to a final concentration of 10 mM. The reaction mixture was stirred at 24°C and 650 rpm for 4 h. For analysis, 1 mL of the reaction mixture was taken, and sodium chloride (100 mg) and 1 mL of pentane containing 1‐adamantanol (8 mM) as an internal standard were added, and the resulting mixture was shaken and centrifuged (4000 *× g*, 2 min, 4°C) to separate the phases. The extraction was repeated three times. The combined organic phases were dried over Na_2_SO_4_ and analyzed by GC‐MS.

### 3.8 Up‐Scaled Procedure for Biotransformation

BAD lyophilisate (1.6 g) was rehydrated in 90 mL potassium phosphate buffer (100 mM, pH = 7) by stirring at 650 rpm at 24°C for 20 min. The substrate was added to the reaction mixture to a final concentration of 10 mM as a solution in 10 mL of 2‐propanol. The reaction mixture was stirred at 24°C and 650 rpm for 4 h. Sodium chloride (5 g) was added, and the reaction mixture was further stirred for 10 min. The mixture was extracted with pentane (3 × 40 mL), and the suspension was separated by centrifugation. The combined organic phases were dried over Na_2_SO_4_ and analyzed by GC‐MS. After evaporation of the organic phase to dryness, the products were purified by column chromatography on silica gel (eluent – pentane/ether changed gradually: 10/1, 7/3, 1/1) to isolate the major components. The respective fractions were combined, concentrated in vacuum, and ^1^H and ^13^C NMR spectra of the products were compared with those of authentic reference samples.

### 3.9 Dess‐Martin Oxidation of (*Z*)‐2‐nonen‐1‐ol (**1i**)

To a solution of (*Z*)‐2‐nonen‐1‐ol (**1i**) (650 mg, 4.57 mmol) in dichloromethane (100 mL), Dess‐Martin periodinane (3.88 g, 9.15 mmol) was added in one portion. The reaction mixture was stirred at RT for 5 h, quenched with 20% aq. Na_2_SO_3_ (10 mL) and with saturated aq. NaHCO_3_ (80 mL). The mixture was stirred until the aq. layer was clear. The aq. layer was extracted with DCM (3 × 30 mL). The combined organic phases were dried (Na_2_SO_4_) and concentrated in vacuum [39].

### 3.10 Catalytic Activity of Lyophilisates of *P. sapidus* and *P. eringii*

Lyophilisates of the fungi were prepared according to the procedure described in 2.3. The corresponding lyophilisate (320 mg) was rehydrated in 18 mL potassium phosphate buffer (100 mM, pH = 7) by stirring at 650 rpm at 24°C for 10 min. The substrate was added to the reaction mixture as a solution in 2 mL of 2‐propanol to a final concentration of 10 mM. The reaction mixture was stirred at 24°C and 650 rpm for 4 h. For analysis, 1 mL of the reaction mixture was taken, sodium chloride (100 mg) was added, and the reaction mixture was shaken. 1 mL of pentane containing 1‐adamantanol (8 mM) as internal standard was added, and the resulting mixture was shaken and centrifuged (4000 *× g*, 2 min, 4°C) to separate the phases. The extraction was repeated three times. The combined organic phases were dried over Na_2_SO_4_ and analyzed by GC‐MS.

## 4 Conclusions

In conclusion, an oxidation protocol for allylic primary alcohols that utilizes fungal lyophilisates as a source of stable oxidative enzymes, aqueous media with 10% isopropanol as a green cosolvent, and atmospheric oxygen as an oxidant was developed. The approach relies on mild conditions and allows obtaining target aldehydes, avoiding overoxidation as well as reduction of activated CC‐double bonds. While the retention of configuration was observed for *(E)‐*configured alcohols, the (*Z*)‐forms were isomerized to the more stable *(E)‐*diastereomers. No products of oxidation were observed for the tested non‐primary allylic alcohols, namely 1‐hepten‐3‐ol, 1‐nonen‐3‐ol, 1‐octyn‐3‐ol, and cyclohexen‐1‐ol. The reaction times are short, the workup is simple, and the concentration of the substrate reaches 5 mM of substrate for 1.6 g of lyophilisate. Continued efforts will aim to broaden the substrate scope, including more complex primary alcohols and secondary allylic alcohols with diverse structures. Additionally, exploring immobilization strategies or the reuse of the lyophilisate may enhance the economic and practical value of the method. Given its high selectivity, mild reaction conditions, and environmentally friendly nature, this approach holds promise as a sustainable alternative for industrial production of flavor and fragrance aldehydes, especially where diastereoselectivity is important.

## Author Contributions

**Letizia Calza**: visualization, investigation, and methodology. **Valeriia Nikitenkova**: visualization, investigation, methodology, and writing – review and editing. **Wendell Albuquerque**: methodology. **Holger Zorn**: resources and writing – review and editing. **Tatyana Zhuk**: conceptualization, methodology, formal analysis, supervision, writing – original draft, and review and editing.

## Conflicts of Interest

The authors declare no conflicts of interest.

## Supporting information




**Supporting File 1**: cbdv70364‐sup‐0001‐SuppMat.pdf

## Data Availability

The authors confirm that the data supporting the findings of this study are available within the article and its Supporting Information section.
