# Supplementary material for: Selective Oxidation of α,β‐Unsaturated Alcohols With Lyophilisates of Bjerkandera adusta
Source: Chem Biodivers. 2025 Aug 18;22(12):e01127. doi: 10.1002/cbdv.202501127 (PMC12716018; doi:10.1002/cbdv.202501127)
Supplement: Supplementary file 1 — Supporting File 1: cbdv70364‐sup‐0001‐SuppMat.pdf [file CBDV-22-e01127-s001.pdf]

Supporting Information

Table of content

Details of the transformations with BAD lyophilisate S3

Characterization data of products S5

NMR spectra of isolated products S7

References S14

Details of the transformations with BAD lyophilisate

Biotransformation of (*E*)*-2-octen-1-ol* (**1a**)

According to the general procedure, **1a** (1 mmol, 152 μL) was added as a solution in 10 mL of 2-propanol to a lyophilisate of *B. adusta* after 20 min of its rehydration in phosphate buffer. The reaction mixture was stirred for 4 h. Purification of the crude gave 109 mg of (*E*)-2-octenal (**2a**, 87%) as a pale yellow colorless liquid. ^{1}H and ^{13}C spectra were identical to those of the authentic reference standard.

Biotransformation of (*E*)*-2-hexen-1-ol* (**1d**)

According to the general procedure, **1d** (1 mmol, 119 μL) was added as a solution in 10 mL of 2-propanol to a lyophilisate of *B. adusta* after 20 min of its rehydration in phosphate buffer. The reaction mixture was stirred for 4 h. Purification of the crude gave 20 mg of (*E*)-2-hexenal (**2d**, 20%) as a colorless liquid. ^{1}H and ^{13}C spectra were identical to those of the authentic reference standard.

Biotransformation of (*E,E*)*-2,4-hexadien-1-ol* (**1e**)

According to the general procedure, **1e** (1 mmol, 98 mg) was added as a solution in 10 mL of 2-propanol to a lyophilisate of *B. adusta* after 20 min of its rehydration in phosphate buffer. The reaction mixture was stirred for 4 h. Purification of the crude gave 62 mg of (*E,E*)-2,4-hexadienal (**2e**, 65%) as a colorless liquid. ^{1}H and ^{13}C spectra were identical to those of the authentic reference standard.

Biotransformation of (*E,E*)*-2,4-decadien-1-ol* (**1f**)

According to the general procedure, **1f** (1 mmol, 154 mg) was added as a solution in 10 mL of 2-propanol to a lyophilisate of *B. adusta* after 20 min of its rehydration in phosphate buffer. The reaction mixture was stirred for 4 h. Purification of the crude gave 103 mg of (*E,E*)-2,4-

decadienal (**2f**, 68%) as a yellow liquid. ^{1}H and ^{13}C spectra were identical to those of the authentic reference standard.

Biotransformation of geraniol (**1g**)

According to the general procedure, **1g** (1 mmol, 173 μL) was added as a solution in 10 mL of 2-propanol to a lyophilisate of *B. adusta* after 20 min of its rehydration in phosphate buffer. The reaction mixture was stirred for 4 h. Purification of the crude gave 144 mg of citral (**2g**, 68%) as a pale yellow liquid. ^{1}H and ^{13}C spectra were identical to those of the authentic reference standard.

Biotransformation of nerol (**1h**)

According to the general procedure, **1h** (1 mmol, 173 μL) was added as a solution in 10 mL of 2-propanol to a lyophilisate of *B. adusta* after 20 min of its rehydration in phosphate buffer. The reaction mixture was stirred for 4 h. Purification of the crude gave 79 mg of citral (**2g**, 68%) as a pale yellow liquid. ^{1}H and ^{13}C spectra were identical to those of the authentic reference standard.

Biotransformation of (*Z*)*-2-nonen-1-ol* (**1i**)

According to the general procedure, **1i** (1 mmol, 168 μL) was added as a solution in 10 mL of 2-propanol to a lyophilisate of *B. adusta* after 20 min of its rehydration in phosphate buffer. The reaction mixture was stirred for 4 h. Purification of the crude gave 116 mg of (*E*)-2-nonenal (**2b**, 83%) as a colourless liquid. ^{1}H and ^{13}C spectra were identical to those of the authentic reference standard.

Characterization data of products

(*E*)-2-octenal (2a): ^{1}H NMR (400 MHz, $CDCl_3$, ppm): δ = 9.50 (d, J = 7.8 Hz, 1H), 6.86 (dt, J = 6.8, J = 15.4 Hz, 1H), 6.11 (ddt, J = 1.6, J = 7.6, J = 15.5 Hz, 1H), 2.36-2.29 (m, 2H), 1.52-1.39 (m, 2H), 1.35-1.19 (m, 4H), 0.89 (m, 3H). ^{13}C NMR (100 MHz, $CDCl_3$, ppm): δ = 194.33 (CHO), 159.01 (C=C), 132.99 (C=C), 32.66 (CH_2), 31.26 (CH_2), 27.35 (CH_2), 22.35 (CH_2), 13.87 (CH_3). This compound was previously described. [1]

(*E*)-2-hexenal (2d): ^{1}H NMR (400 MHz, $CDCl_3$, ppm): δ = 9.48 (d, J = 8.1 Hz, 1H), 7.10 (m, 1H), 6.32 (m, 2H), 6.06 (m, 1H), 1.92 (d, J = 5.8 Hz, 3H). ^{13}C NMR (100 MHz, $CDCl_3$, ppm): δ = 194.09 (CHO), 158.77 (C=C), 133.12 (C=C), 34.61 (CH_2), 21.08 (CH_2), 13.56 (CH_3). This compound was previously described. [1]

(*E,E*)-2,4-hexadienal (2e): ^{1}H NMR (400 MHz, $CDCl_3$, ppm): δ = 9.54 (d, J = 8.0 Hz, 1H), 6.83 (dt, J = 6.8, J = 15.7 Hz, 1H), 6.09 (ddt, J = 1.4, J = 7.9, J = 15.6 Hz, 1H), 2.33-2.26 (m, 2H), 1.57-1.47 (m, 2H), 0.94 (t, J = 7.6 Hz, 3H). ^{13}C NMR (100 MHz, $CDCl_3$, ppm): δ = 194.04 (CHO), 152.65 (C=C), 141.94 (C=C), 130.13 (C=C), 129.90 (C=C), 18.96 (CH_3). This compound was previously described [2].

(*E,E*)-2,4-decadienal (2f): ^{1}H NMR (400 MHz, $CDCl_3$, ppm): δ = 9.53 (d, J = 8.2 Hz, 1H), 7.09 (m, 1H), 6.37-6.24 (m, 2H), 6.07 (dd, J = 8.2, J = 15.3 Hz, 1H), 2.25-2.18 (m, 2H), 1.51-1.42 (m, 2H), 1.39-1.25 (m, 4H), 0.90 (t, J = 6.8 Hz, 3H). ^{13}C NMR (100 MHz, $CDCl_3$, ppm): δ = 194.08 (CHO), 152.88 (C=C), 147.46 (C=C), 130.27 (C=C), 128.72 (C=C), 33.19 (CH_2), 31.35 (CH_2), 28.22 (CH_2), 22.43 (CH_2), 13.97 (CH_3). This compound was previously described [3].

Citral (2g): ^{1}H NMR (400 MHz, $CDCl_3$, ppm): δ = 9.96-9.87 (m, 1H), 5.85 (m, 1H), 5.06 (m, 1H), 2.56 (t, J = 7.5 Hz, 1H), 2.21 (m, 3H), 2.14 (d, J = 1.4 Hz, 2H), 1.96 (d, J = 1.4 Hz, 1H), 1.66 (s, 3H), 1.57 (d, J = 6.5 Hz, 3H). ^{13}C NMR (100 MHz, $CDCl_3$, ppm): δ = 191.22 (CHO), 190.68 (CHO), 163.77 (2C=C), 133.61 (C=C), 132.83 (C=C), 128.61 (C=C), 127.38 (C=C), 122.59 (C=C), 122.28 (C=C), 40.55 (CH_2), 32.57 (CH_2), 27.05 (CH_2), 25.71 (CH_2), 25.59 ($2CH_3$), 25.03 (CH_3), 17.65 (CH_3), 17.53 ($2CH_3$). This compound was previously described [4].

(*E*)-2-nonenal (2b): ^{1}H NMR (400 MHz, $CDCl_3$, ppm): δ = 9.49 (d, J = 7.8 Hz, 1H), 6.83 (dt, J = 6.9, J = 15.6 Hz, 1H), 6.11 (ddt, J = 1.5, J = 8.0, J = 15.6 Hz, 1H), 2.36-2.29 (m, 2H), 1.51-1.47 (m, 2H), 1.37-1.21 (m, 6H), 0.86 (m, 3H). ^{13}C NMR (100 MHz, $CDCl_3$, ppm): δ = 194.33 (CHO), 159.01 (C=C), 133.01 (C=C), 32.76 (CH_2), 31.55 (CH_2), 28.85 (CH_2), 27.84 (CH_2), 22.53 (CH_2), 13.99 (CH_3). This compound was previously described [5].

(*Z*)-2-nonenal (1n): ^{1}H NMR (400 MHz, $CDCl_3$, ppm): δ = 10.08 (d, J = 8.5 Hz, 1H), 6.67 (dt, J = 8.2, J = 11 Hz, 1H), 5.96 (m, 1H), 2.61 (m, 1H), 1.57-1.47 (m, 3H), 1.40-1.19 (m, 6H), 0.92-0.86 (m, 3H). ^{13}C NMR (100 MHz, $CDCl_3$, ppm): δ = 191.23 (CHO), 153.64 (C=C), 130.31 (C=C), 31.53 (CH_2), 29.19 (CH_2), 28.75 (CH_2), 28.01 (CH_2), 22.51 (CH_2), 14.00 (CH_3). This compound was previously described [6].

NMR spectra of isolated products

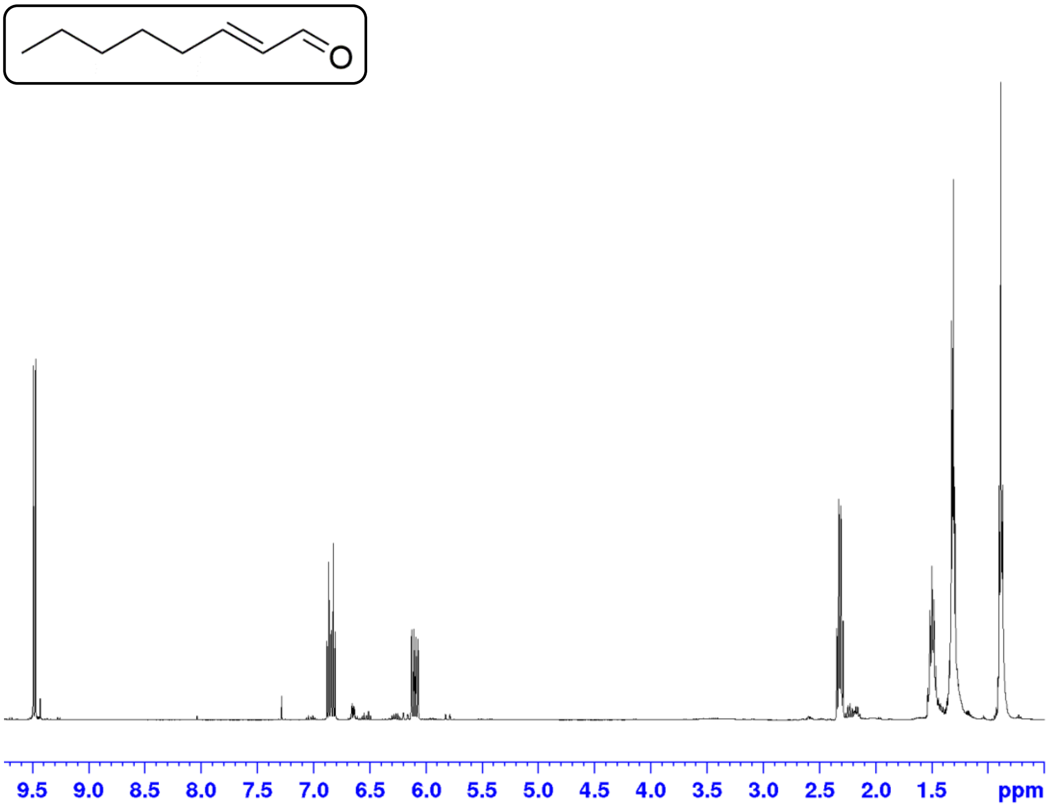

Figure S1. ^{1}H NMR of (*E*)-2-octenal (**2a**).

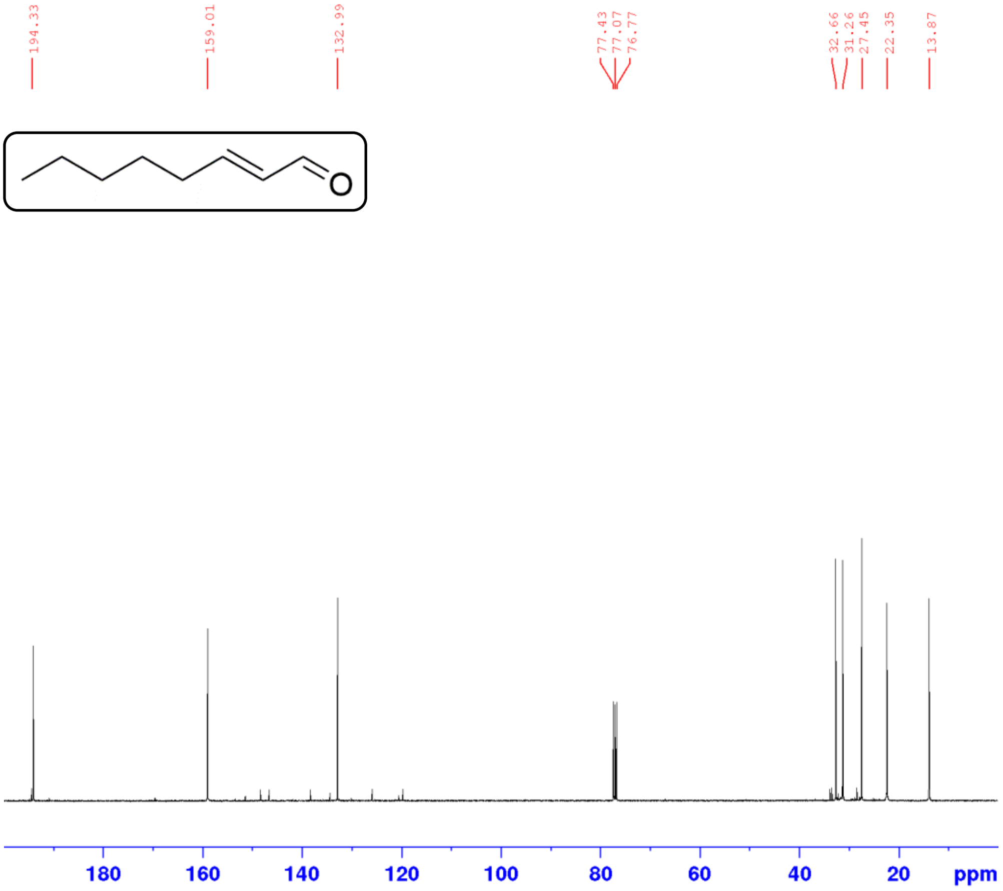

Figure S2. ^{13}C NMR of (*E*)-2-octenal (**2a**).

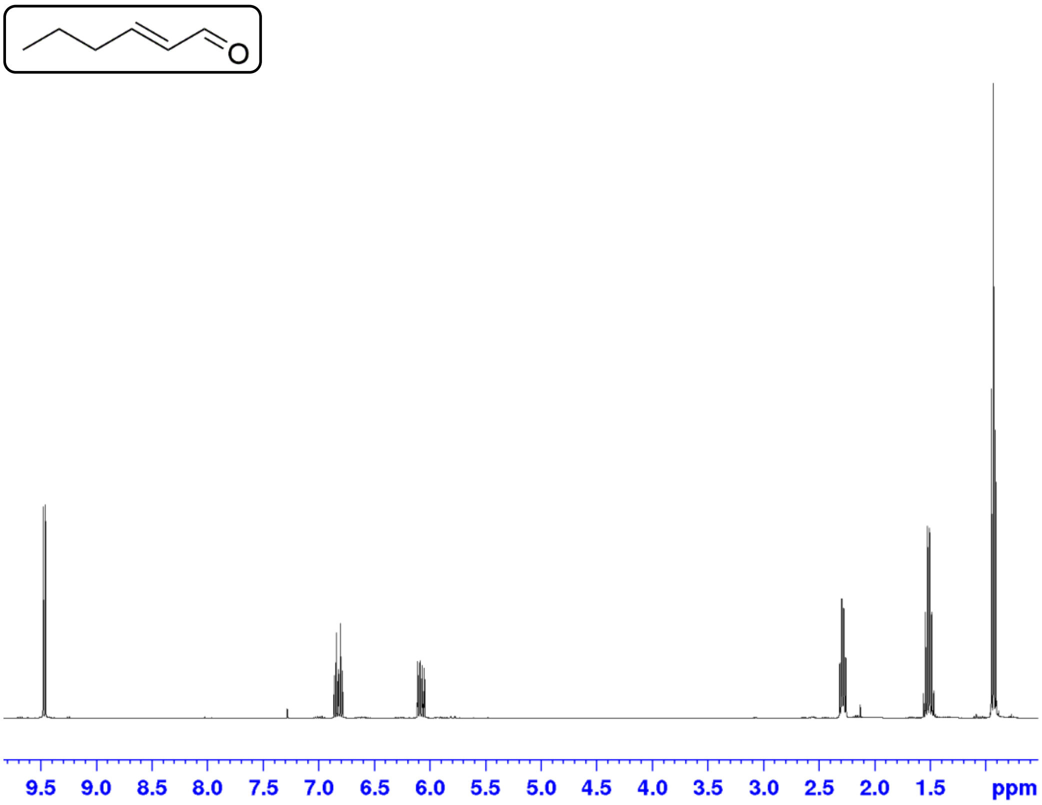

Figure S3. ^{1}H NMR of (*E*)-2-hexenal (**2d**).

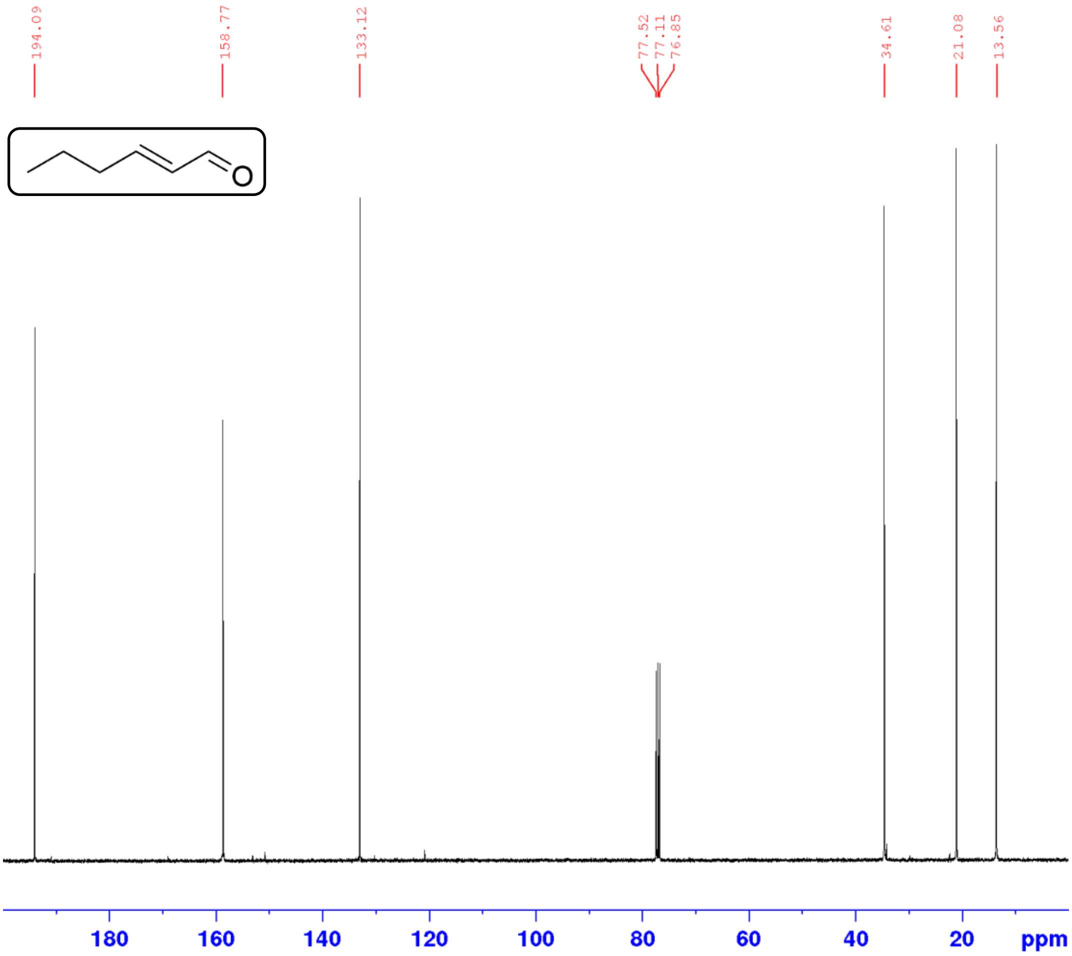

Figure S4. ^{13}C NMR of (*E*)-2-hexenal (**2d**).

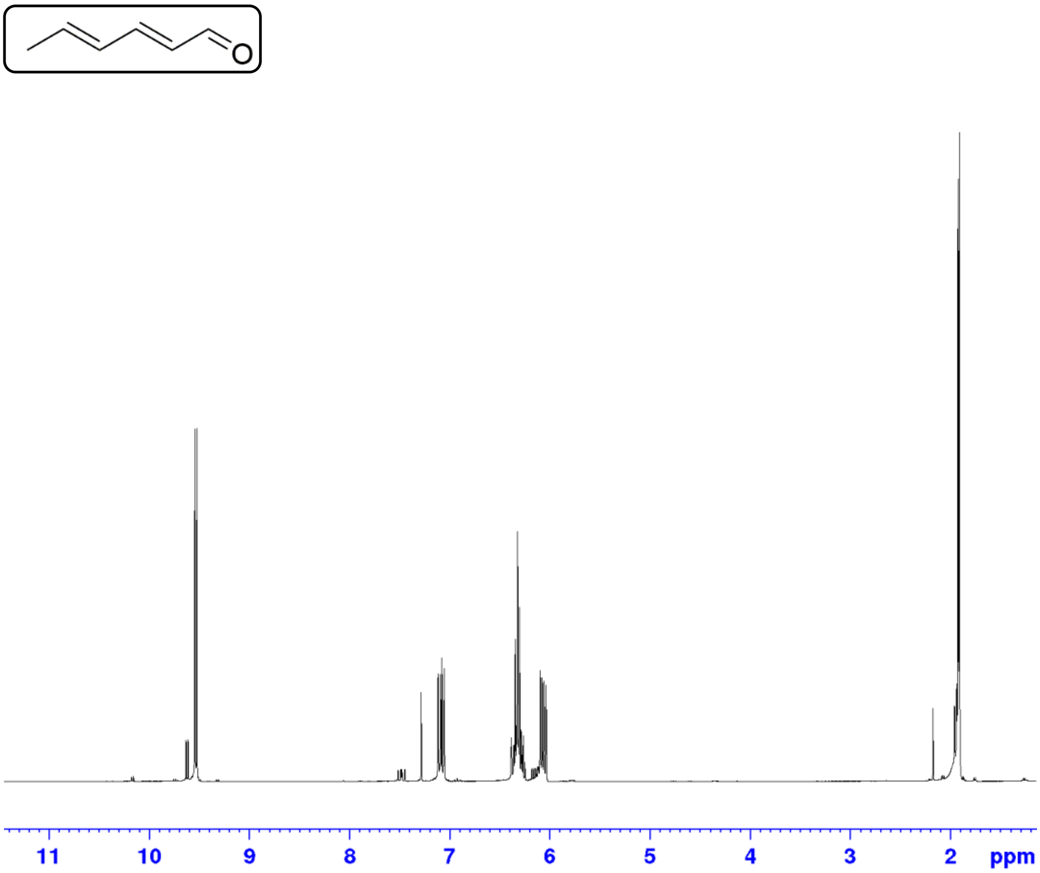

Figure S5. ^{1}H NMR of (*E*,*E*)-2,4-hexadienal (**2e**).

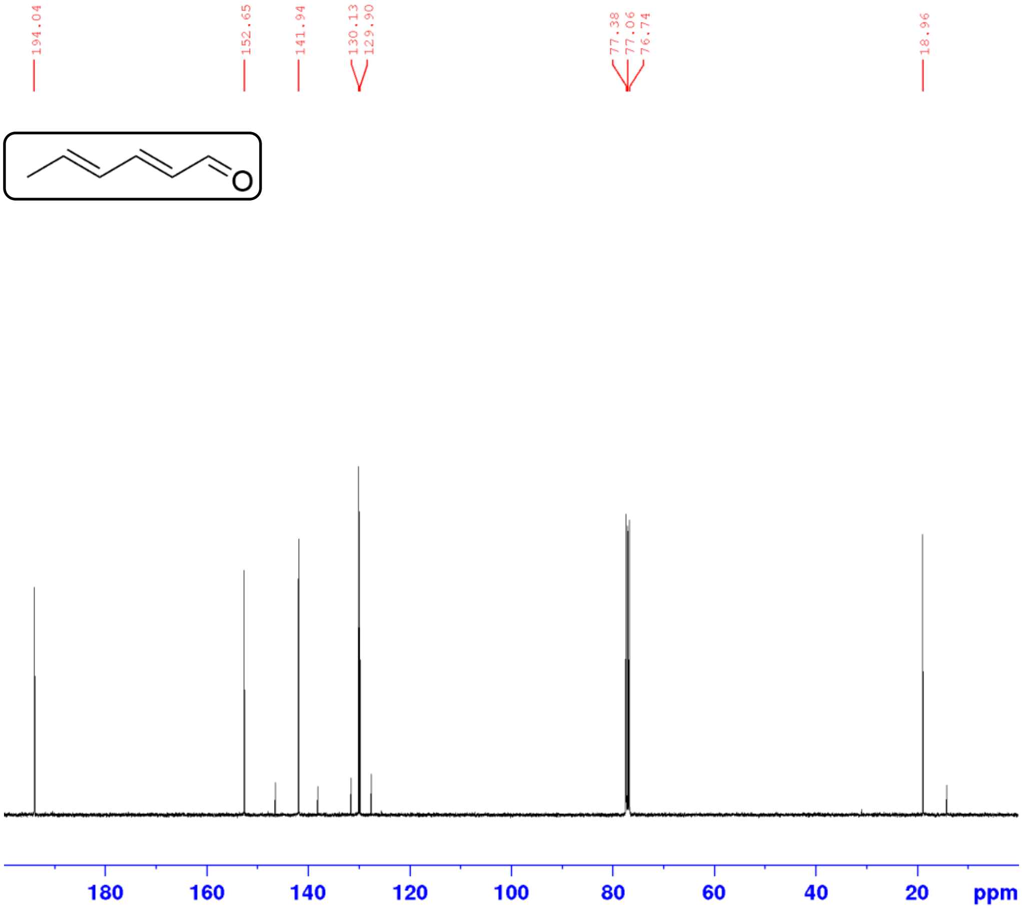

Figure S6. ^{13}C NMR of (*E*,*E*)-2,4-hexadienal (**2e**).

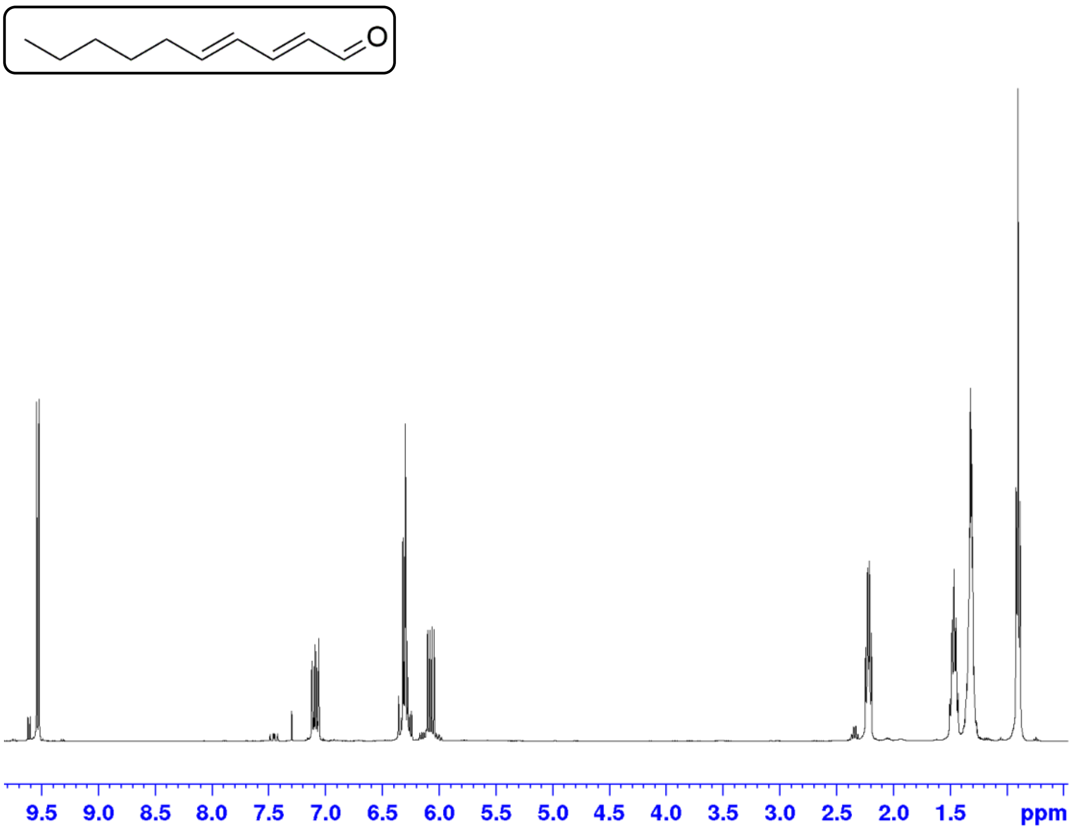

Figure S7. ^{1}H NMR of (*E*,*E*)-2,4-decadienal (**2f**).

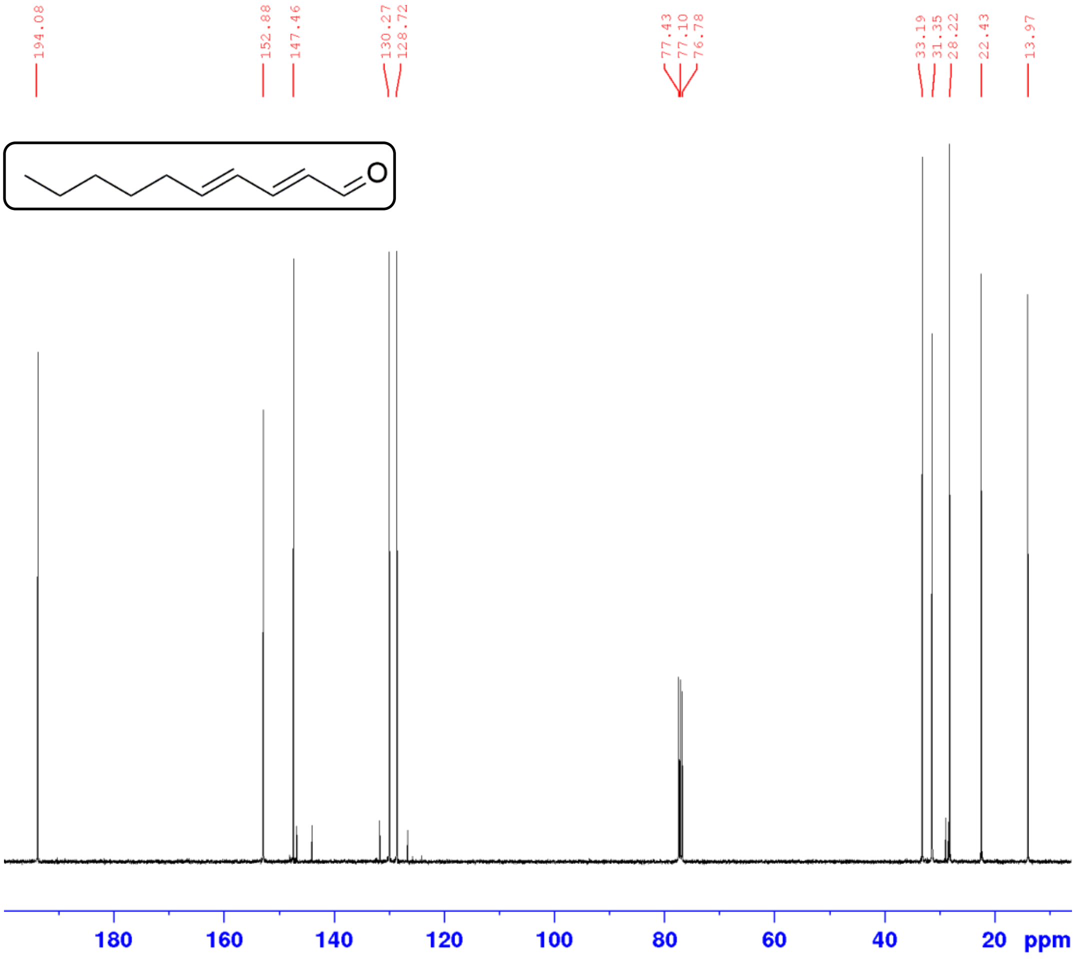

Figure S8. ^{13}C NMR of (*E*,*E*)-2,4-decadienal (**2f**).

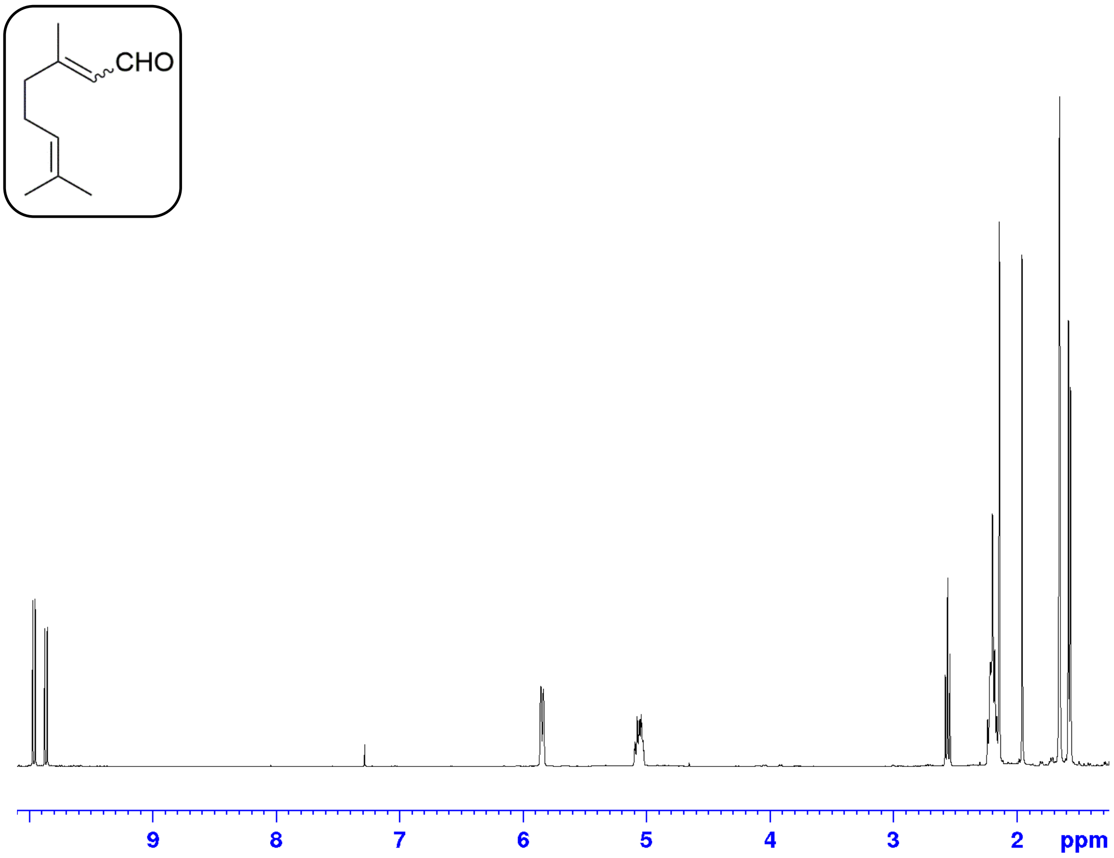

Figure S9. ^{1}H NMR of citral (**2g**).

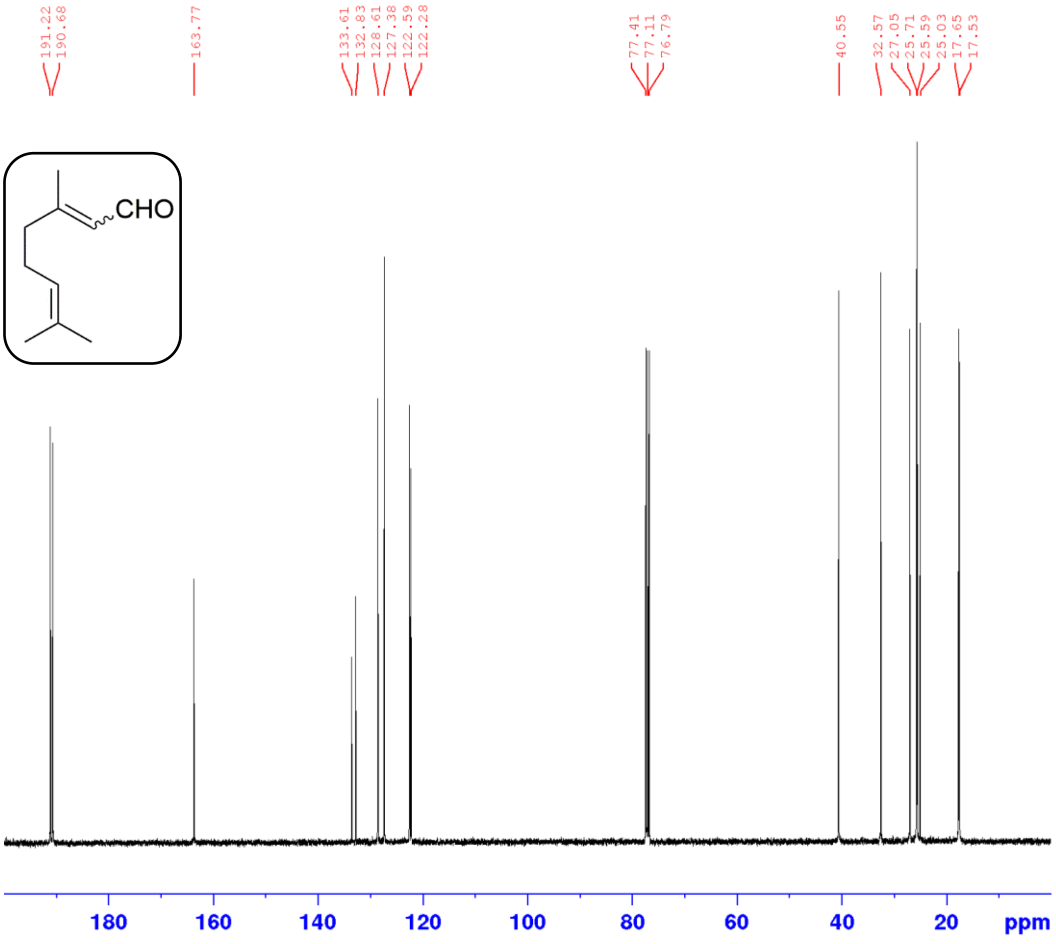

Figure S10. ^{13}C NMR of citral (**2g**).

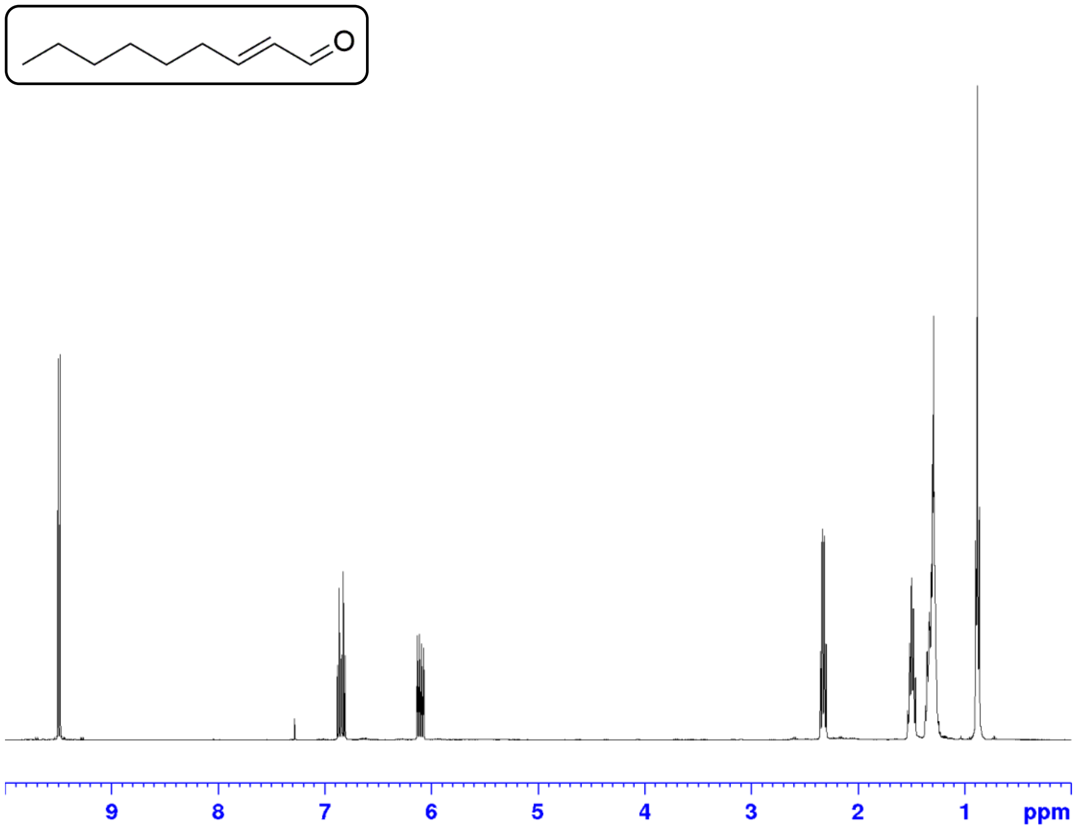

Figure S11. ^{1}H NMR of (*E*)-2-nonenal (**2b**).

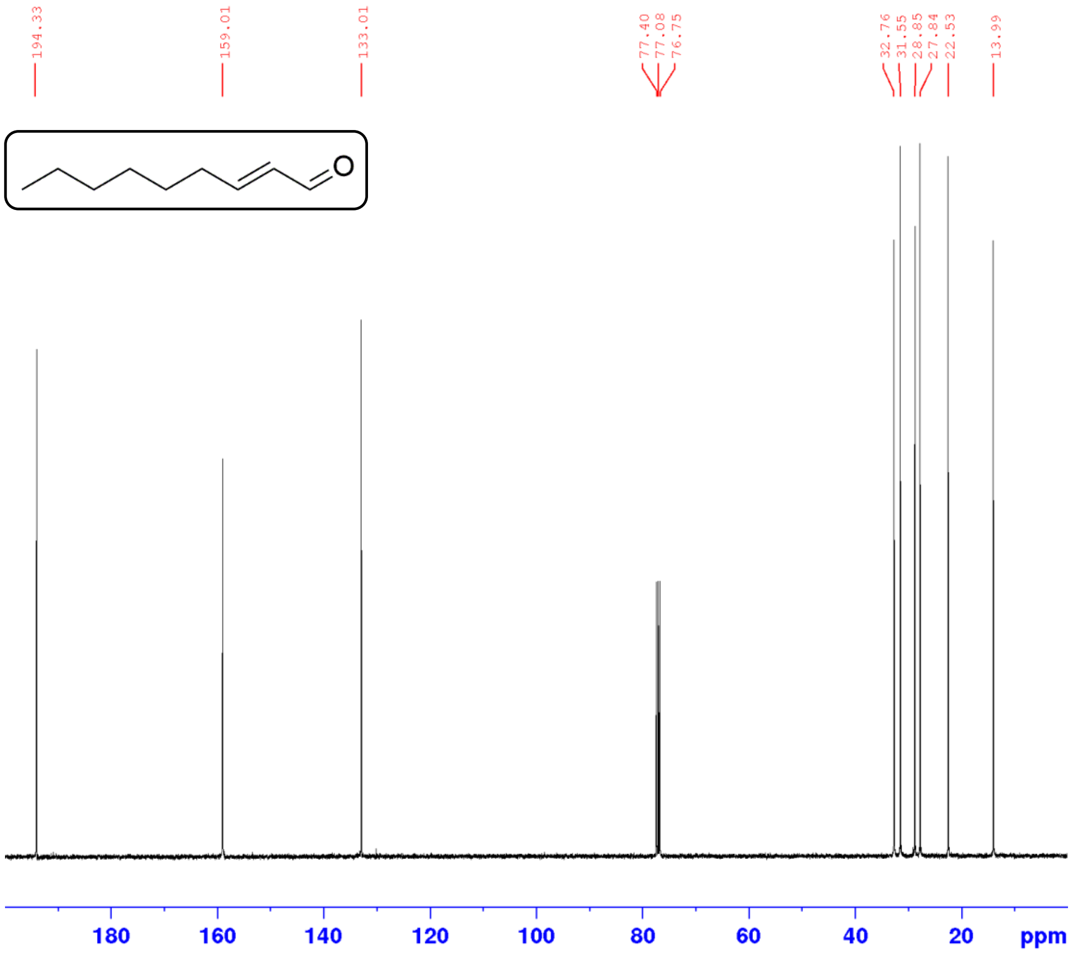

Figure S12. ^{13}C NMR of (*E*)-2-nonenal (**2b**).

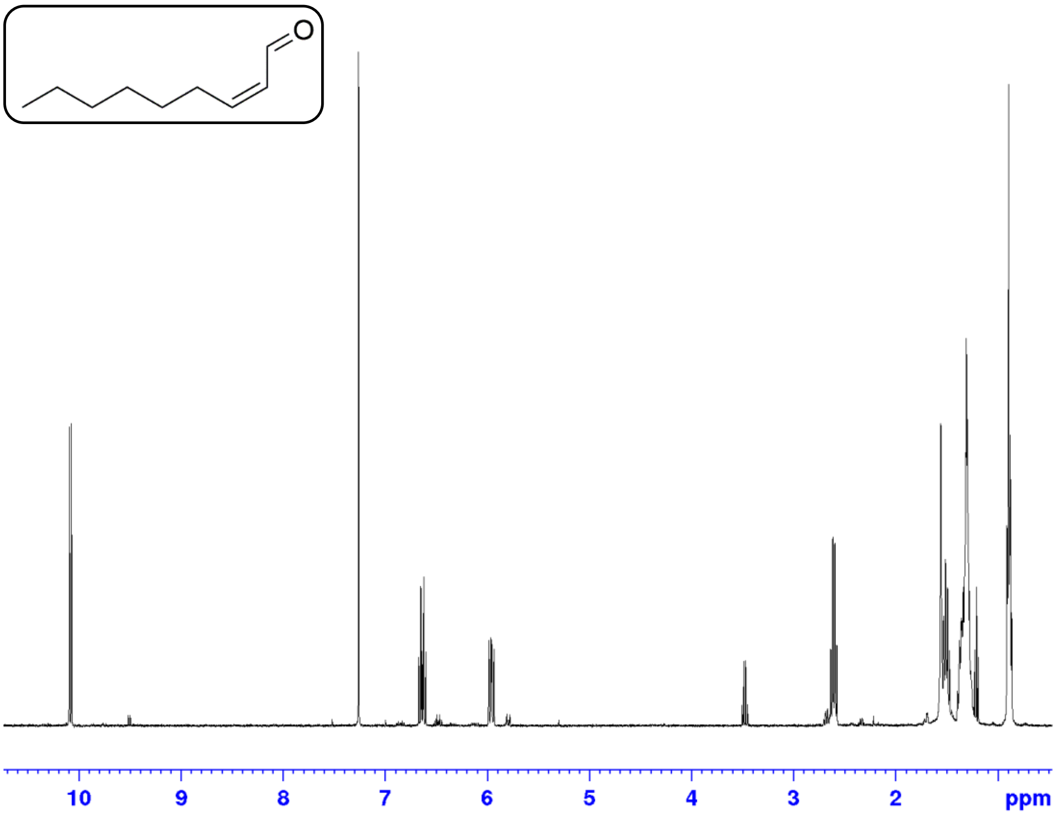

Figure S13. ^{1}H NMR of (*Z*)-2-nonenal (**1n**).

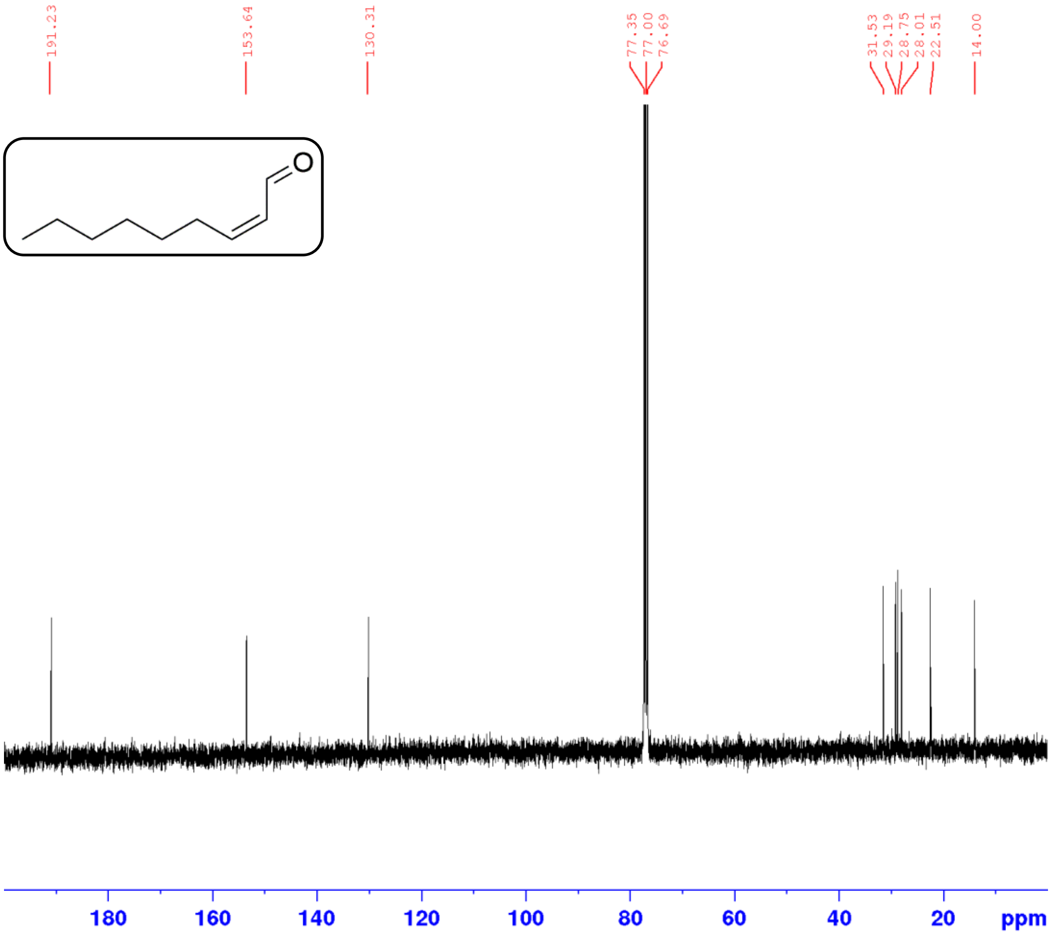

Figure S14. ^{13}C NMR of (*Z*)-2-nonenal (**1n**).

References

[1] Y. Kon; Y. Usui; K. Sato, Oxidation of allylic alcohols to α,β-unsaturated carbonyl compounds with aqueous hydrogen peroxide under organic solvent-free conditions. *ChemComm* **2007,** (42), 4399-4400.

[2] N. Jiang; A. J. Ragauskas, Cu(II)-Catalyzed Selective Aerobic Oxidation of Alcohols under Mild Conditions. *J. Org. Chem.* **2006,** *71* (18), 7087-7090.

[3] G.-F. Pan; X.-L. Zhang; X.-Q. Zhu; R.-L. Guo; Y.-Q. Wang, Synthesis of (E,E)-Dienones and (E,E)-Dienals via Palladium-Catalyzed γ,δ-Dehydrogenation of Enones and Enals. *iScience* **2019,** *20*, 229-236.

[4] M. Guan; C. Wang; J. Zhang; Y. Zhao, Practical organic solvent-free Cu(OAc)2/DMAP/TEMPO-catalyzed aldehyde and imine formation from alcohols under air atmosphere. *RSC Adv.* **2014,** *4* (90), 48777-48782.

[5] Y. Zhu; B. Zhao; Y. Shi, Highly Efficient Cu(I)-Catalyzed Oxidation of Alcohols to Ketones and Aldehydes with Diaziridinone. *Org. Lett.* **2013,** *15* (5), 992-995.

[6] K. Matsumoto; K. Oohana; M. Hashimoto; K. Usuda; T. Shimoda; H. Ohshima; Y. Suzuki; T. Togawa, Enzyme-mediated enantioselective hydrolysis of 1,2-diol monotosylate derivatives bearing an unsaturated substituent. *Tetrahedron* **2018,** *74* (29), 3981-3988.
